# Treatment of Middle Ear Ventilation Disorders: Sheep as Animal Model for Stenting the Human Eustachian Tube – A Cadaver Study

**DOI:** 10.1371/journal.pone.0113906

**Published:** 2014-11-24

**Authors:** Felicitas Miller, Alice Burghard, Rolf Salcher, Verena Scheper, Wolfgang Leibold, Thomas Lenarz, Gerrit Paasche

**Affiliations:** 1 Department of Otolaryngology, Hannover Medical School, Hannover, Germany; 2 Department of Immunology, University of Veterinary Medicine Hannover, Foundation, Hannover, Germany; 3 Hearing4all Cluster of Excellence, Hannover Medical School, Hannover, Germany; Katholieke Universiteit Leuven, Belgium

## Abstract

Eustachian tube disorders can lead to chronic otitis media with consecutive conductive hearing loss. To improve treatment and to develop new types of implants such as stents, an adequate experimental animal model is required. As the middle ear of sheep is known to be comparable to the human middle ear, the dimensions of the Eustachian tube in two strains of sheep were investigated. The Eustachian tube and middle ear of half heads of heathland and blackface sheep were filled with silicone rubber, blended with barium sulfate to induce X-ray visibility. Images were taken by digital volume tomography. The tubes were segmented, and a three-dimensional model of every Eustachian tube was generated. The lengths, diameters and shapes were determined. Additionally, the feasibility of endoscopic stent implantation and fixation was tested in cadaver experiments. The length of the tube between ostium pharyngeum and the isthmus and the diameters were comparable to published values for the human tube. The tube was easily accessible through the nose, and then stents could be implanted and fixed at the isthmus. The sheep appears to be a promising model for testing new stent treatments for middle ear ventilation disorders.

## Introduction

As the only connection from the middle ear to the pharynx, the Eustachian tube (ET) is responsible for ventilation of the middle ear, as well as drainage. If its function is disturbed, this can lead to acute and chronic otitis media, cholesteatoma and consecutive hearing impairment [1], [2]. Today, about 0.9% of the British population is suffering from ventilation disorders of the ET [3] and there is still no effective therapy for many patients affected chronically [4].

Over the years different approaches to treat ET disorders were investigated. One of the first approaches was an expansion of the ET with a polyvinylchlorid (PVC) tube connected to a thread, which stayed in the middle ear for up to 10 days [5]. Later a permanent tube stent made from silastic [6] was tested. This prosthesis had severe adverse effects such as protrusion and extrusion of the stent, as well as acute otitis media and development of granulation tissue, and did not improve the function of the ET [7]. Even treatment with a gold wire that was permanently implanted from the middle ear [8] did not provide the success desired. Reported success rates regarding better middle ear ventilation varied considerably between 8.3% [9] and 66% [10]. Due to lack in success there is a need to develop new and effective treatments for ET disorders [4], [9].

Additionally, balloon dilatation gained attention during the last years [11]–[14]. New cadaver studies [13] and also first clinical trials [12] showed that dilatation of the ET leads to a widening of the tube for up to six month and had no visible side effects or complication other than linear fissuring within the ET lumen [12], [13]. However long term data on clinical success derived from controlled clinical studies is missing.

In order to develop effective treatment modalities for ET dysfunction a valid animal model is required. Gerbil [15], chinchilla [16] and rabbit [17] have been used to investigate certain aspects of an ET treatment. These models are appropriate for biocompatibility tests [17], but even if the overall structure and histology of the tube is comparable to human [18], the size of the ET is much smaller. This leads to several problems such that stents being produced in human size need to be adapted for the respective species and their function can be affected [16]. Adapting the samples to animal size can lead to a different stiffness, stability and biodegradation in comparison to human sized samples. Therefore, an animal model that enables *in vivo* testing of human sized samples and treatment options is appropriate when investigating possible approaches to treat ET malfunctions in humans.

Generally sheep and pig are considered to be a good model for the human middle ear [19] even though the ET was not investigated in this study. Recently, the sheep was found to be also suitable for implantable hearing devices [20]. In contrast, for pigs it was reported earlier that the bony part of the ET is missing and the entire tube consists of a cartilaginous structure [21]. Therefore, the aim of the current cadaver study was to investigate whether the ET of sheep can be a model for the human ET in terms of dimensions and feasibility for surgical procedures such as balloon dilatation and stent implantation.

## Materials and Methods

### Sample preparation

As this study was a cadaver study using thirteen fresh frozen heads of heathland sheep (Landschlachterei Hermann Meyer e.K., Bispingen, Germany) and six of blackface sheep (Hencke Fleischwaren GmbH, Bad Bevensen, Germany) as received from slaughterhouses ethics committee approval was not required. After cutting in the median plane heads were defrosted, the outer ears were removed and the middle ears were opened.

The Eustachian tube was filled through the opened middle ear with a 2-component silicone rubber (Smooth-Sil 940 with PlatCat/1 accelerator, SMOOTH-ON, Easton, USA), blended with 20% barium sulfate for an enhanced X-ray contrast. To achieve this concentration, component one of the silicone was mixed at room temperature with 30% barium sulfate. Then component two was added at a ratio of 1∶13 before 2% of the accelerator was added resulting in a final BaSO_4_ concentration of about 20%. To increase the viscosity of the silicone the barium sulfate content was later raised as needed to prevent the semiliquid silicone from leaking out of the ostium pharyngeum. Using this approach, the ET of 10 half heads of heathland sheep and 6 of blackface sheep were filled with silicone. After 24 hours of drying at room temperature the heads were frozen again. The length of the cured silicone outside the ostium pharyngeum was determined by means of a caliper.

### Imaging

**Figure 1:**
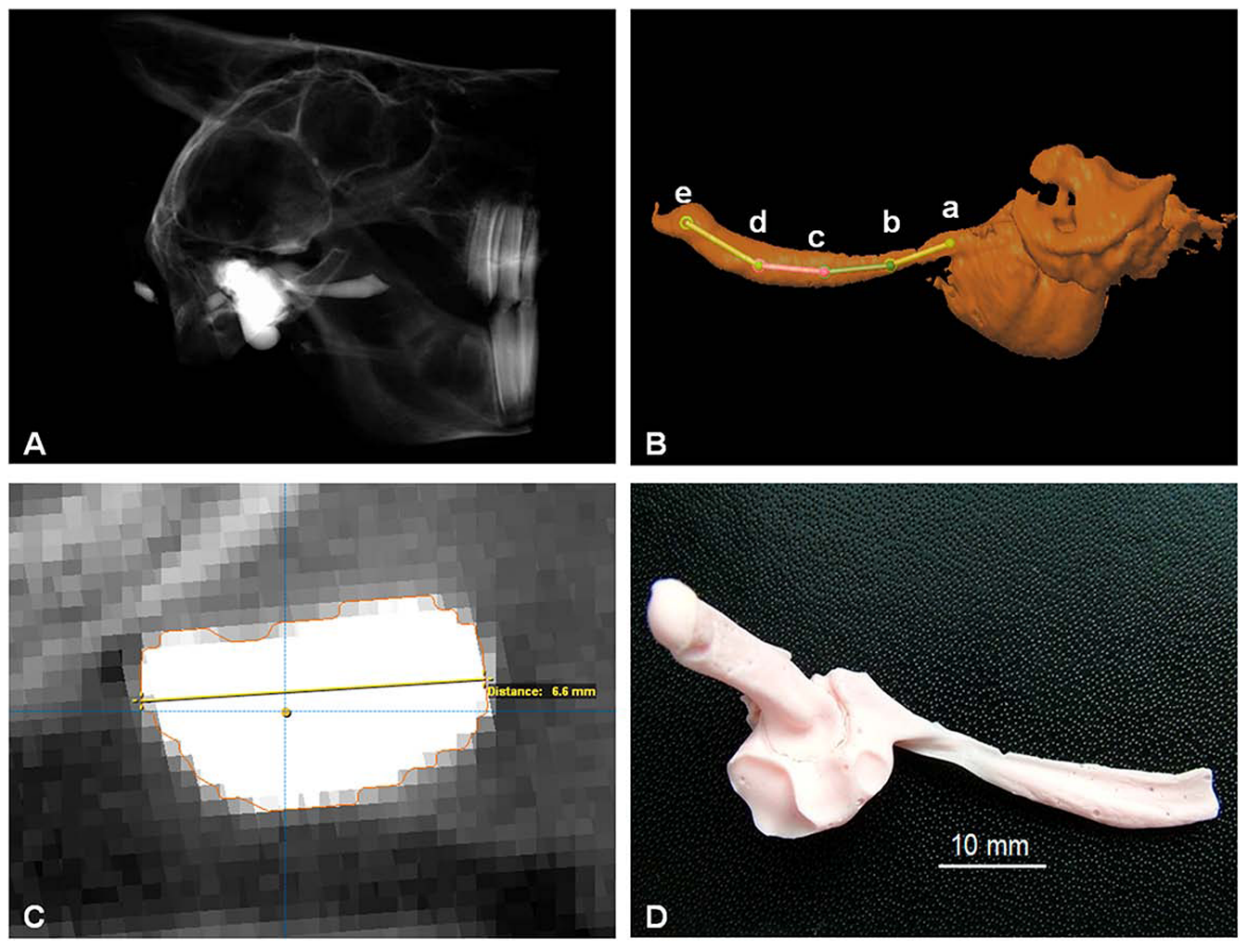
Determination of the dimensions of the Eustachian tube in heathland sheep. **A**: DVT scan. **B**: segmented tube and middle ear from DVT scan data. The trajectories in the centre of the tube are indicated together with the start and end points (a–e) of each trajectory. **C**: Cross section of a segmented tube. The dot in the centre marks the position of the trajectory. Perpendicular to it, the diameters were measured. The indicated line refers to a length of 6.6 mm for the larger diameter. **D**: Macerated silicone model of the tube including (from right to left) tube, middle ear and parts of the outer ear canal.

Three-dimensional images of the frozen heads were taken by digital volume tomography (DVT) (xCAT ENT; Xoran Technologies, Ann Arbor, USA) at 120 KVp and a resolution of 300 µm in all three dimensions (Fig. 1A).

Images were analyzed by the software iPlan 2.6 using PatXfer 5.2 (both BrainLAB AG, Feldkirchen, Germany) and a three-dimensional model of each ET and middle ear was created using the threshold segmentation method. The chosen thresholds were the upper 75% of grey values for heathland sheep and 50% of grey values for blackface sheep. The ostium tympanum was defined as the region where the tubular lumen expands to the wide lumen of the middle ear. The ostium pharyngeum was determined by subtracting the length of the silicone outside the ostium that was measured before.

The ET model was then divided in 4 sections, each with the same number of images. The center of the first and the last sectional image of the ET were marked as start- or endpoint of a trajectory (Fig. 1B). The length of each trajectory was determined and the height and width of the tube model were measured perpendicular to these trajectories every 2 mm (Fig. 1C). After determining the isthmus (smallest diameter along the ET), this region was measured again every millimeter.

Black spots appeared in some images and indicated air bubbles trapped in the silicone. If these black spots where completely surrounded by segmented material they were treated as part of the silicone and also segmented, otherwise excluded.

Furthermore the angles between two neighboring trajectories in the tube model were determined (Fig. 1B, angles a–b–c; b–c–d; c–d–e), and additionally the angle between the centers of the ostium pharyngeum and the ostium tympanum (Fig. 1B; angle between a–c–e) was measured.

### Maceration

To compare the computed dimensions of the tube with the actual ones of the silicone model of the tube, the heathland sheep heads were trimmed to get a small block of tissue containing the filled tube and then macerated with 30% caustic potash (made from H_2_O_2_ 30% and Potassium hydroxide, both Merck KGaA, Darmstadt, Germany). The remaining silicone specimens (Fig. 1D) were measured with a caliper in lengths and diameters at the ostium pharyngeum, the ostium tympanicum, and the isthmus.

### Endoscopy and stent insertion

Experiments were done in the remaining half heads but also in additional 4 full heads. The feasibility of an endoscopic examination of the ET in sheep and the placement of a stent was investigated with a flexible Storz bronchofiberscope for cats (KARL STORZ GmbH & Co. KG, Tuttlingen, Germany; diameter: 3.7 mm; length: 54 cm). Through the working channel of the bronchofiberscope (diameter: 1.5 mm) a flexible miniature optic (diameter: 0.5 mm) could be inserted.

Furthermore, commercial cobalt chromium coronary stent systems (PRO-Kinetic Energy, BIOTRONIK, Bülach, Switzerland) with diameters of 2.0 mm and a length of 9 mm or 20 mm were used for stent insertions with a coronary guide wire (GALEO, BIOTRONIK). The guide wire was inserted into the ET through the working channel of the bronchofiberscope. The stent system was then introduced over the guide wire. The balloon catheter was inflated with a pressure of approximately 12 bar for one minute to expand the stent and deflated afterwards before balloon and guide wire were removed carefully. The positioning of the stent inside the tube was verified by DVT at the same settings as above.

### Statistics

Comparison of dimensions of the macerated silicone tube model with the data generated during segmentation of the DVT scans was done by using paired t-tests.

## Results

### Dimensions

The ET of heathland sheep and blackface sheep could be filled with silicone without significant leaking of the silicone from the ostium pharyngeum. The filled ET was easily detectable in DVT data sets as well as in macerated silicone tube models (Fig. 1).

#### Heathland sheep

Diameters of silicone samples were smaller than the calculated ones from DVT data and all lengths were larger. The average length of the ET was determined to be 27.4 mm±1.3 mm in DVT data and 30.4 mm±2 mm directly at the silicone models (Table 1). The total length comprised the sections from the middle ear to the isthmus (DVT: 5.8 mm±1.5 mm; silicone: 7.5 mm±2.1 mm) and from the isthmus to the throat (DVT: 21.6 mm±1.6 mm; silicone: 22.9 mm±1.8 mm). The isthmus width was 1.1 mm±0.2 mm in DVT data and 0.5 mm±0.1 mm for silicone models. The differences in measurements of DVT data and silicone models were always significant. P-values are given in Table 1.

**Table 1:** Measured dimensions for the tube of heathland sheep and blackface sheep.

	Heathland sheep					Blackface sheep				
	DVT (N = 10)			Silicone model of ET (N = 10)		DVT (N = 6)			Silicone model of ET (N = 6)	
	Average [mm]	Spread [mm]	p-value	Average [mm]	Spread [mm]	Average [mm]	Spread [mm]	p-value	Average [mm]	Spread [mm]
**Length of ET**	27.4	25.8–29.8	0.0006	30.4	27.1–33.6	28.8	27.3–31.2	0.0079	31.1	28.9–32.4
**Distance middle ear – isthmus**	5.8	3.5–7.5	0.0353	7.5	4–10.9	4.1	0.8–10.7	0.135	6.3	2.1–17.9
**Distance isthmus – throat**	21.6	18.9–24.4	0.0143	22.9	19.5–25	24.7	19.1–27.3	0.916	24.8	13.7–29.1
**Width isthmus**	1.1	0.8–1.5	1.6E-5	0.5	0.4–0.8	1.1	1–1.2	0.0016	0.7	0.4–1.1
**Height isthmus**	3.6	2.3–5.9				3.5	2.7–4.4			
**Diameter ostium pharyngeum**	3.8	2.6–5.6	1.4E-5	1.9	0.8–3.3	3.6	1.9–4.3	0.0934	4.1	3.2–4.5

Due to the small width at the isthmus, reliable measurements of the height of the silicone models were not feasible.

Min and max values (spread) are given instead of the standard deviation. The p-values refer to the respective comparison between DVT data and the silicone model. DVT – digital volume tomography. ET – Eustachian tube.

The average length of the 4 segments of each ET DVT data set was with values between 6.7 mm (c–d) and 7.0 mm (d–e) fairly constant, the angles between two neighboring segments increased from the ostium pharyngeum (e) towards the middle ear (a) from 164.1°±3.2° (mean ± SD) (c–d–e) to 170.6°±3.2° (a–b–c) (Table 2). The angle between the first and last segments (a–c–e) was determined to be 153.2°±5.8°.

**Table 2:** Curvature of the tube.

	Length a–b	Angle a–b–c	Length b–c	Angle b–c–d	Length c–d	Angle c–d–e	Length d–e	Angle a–c–e
HS 1 R	6,4	164,7	6,7	164,5	7	163,5	7,8	152
HS 3 L	6,3	172	6,4	169,8	6,4	162,7	6,7	158,3
HS 6 L	6,5	172,5	6,6	171,4	6,7	158,9	7,3	157,6
HS 6 R	7	170,6	6,5	172,8	6,3	162,8	6,1	160
HS 7 L	7,1	172,9	7,3	157,3	7,5	162,4	7,9	146,9
HS 7 R	7,5	165,2	7,4	167,2	6,9	169	6,7	158,4
HS 8 L	6,9	174	6,8	160,7	6,8	167,8	6,7	153,2
HS 8 R	7,3	171,3	6,9	165,7	6,4	167,4	6,2	156
HS 9 L	7,2	170,3	7	159,5	6,9	161	7,6	145,4
HS 9 R	6,6	172,9	6,7	153,8	6,2	165,4	6,7	144,4
**mean**	**6.9**	**170.6**	**6.8**	**164.3**	**6.7**	**164.1**	**7.0**	**153.2**
BS 1	7.4	173.8	7.3	172.8	7.3	170.5	7.2	169.5
BS 2	6.8	171.9	7	170.9	7.1	168.1	7	163.3
BS 3	7.7	173.7	8	159.3	7.1	158	7	152
BS 4	7.9	172.8	7.9	166.5	7.8	173	7.6	163.7
BS 5	6.5	167.2	6.5	174.5	6.7	159.2	7.6	157.7
BS 6	4.7	165	7.4	175	7.6	175.2	7.6	167.9
**mean**	**6.8**	**170.7**	**7.4**	**169.8**	**7.3**	**167.3**	**7.3**	**162.4**

The angles [°] between the different segments of the tube and the lengths [mm] of these segments are given according to evaluation of DVT data sets.

HS – heathland sheep; BS – blackface sheep; R – right side; L – left side.

#### Blackface sheep

The measures of the ET in blackface sheep (Table 1) were again determined by evaluation of the DVT scan data and macerated tube models. As in heathland sheep all measured lengths of the silicone model were larger than in DVT scan data and – except for the diameter at the ostium pharyngeum – diameters were smaller, but differences were only significant for the total length of the ET and the width of the isthmus (Table 1). The total length of the tube is with 28.8 mm±1.6 mm (DVT) or 31.1 mm±1.4 mm (silicone) slightly larger than in heathland sheep. Whilst all diameters were comparable between both strains, the main difference was found in the distance from the isthmus to the ostium pharyngeum. The average distance in blackface sheep is with 24.7 mm±3 mm (DVT) and 24.8 mm±5.8 mm (silicone) 2 to 3 mm larger than in heathland sheep (DVT: 21.6 mm±1.6 mm; silicone: 22.9 mm±1.8 mm) and very close to the reported values for the human ET (Table 3).

**Table 3:** Dimensions of the human Eustachian tube as given in the literature.

	Bezold [22]		Pahnke [1], [23]		Proctor [24], [25]	
	average	spread	average	spread	average	spread
**Total length of ET**	36.4	34–40	36.2	31.2–42.4		
**Middle ear – isthmus**	13.4	8.5–17.5	7.6	4.2–11.2		11–12
**Isthmus – throat**	24.6	21.5–27			25	24–25
**Width isthmus**		0.2–1.5			1	
**Height isthmus**	3	2–4.5	2.9	1.3–4.4	2	
**Height ostium pharyngeum**						8–9
**Width ostium pharyngeum**						4–5
**Diameter ostium pharyng.**			7.3	6–10		

All values are given in millimeter. ET – Eustachian tube.

The average length of the 4 segments of each ET DVT data set was with values between 6.8 mm (a–b) and 7.4 mm (b–c) slightly larger than in heathland sheep. The angles between two neighboring segments increased from the ostium pharyngeum towards the middle ear from 167.3°±7.2° (mean ± SD) (c–d–e) to 170.7°±3.7° (a–b–c) (Table 2). The angle between the first and the last segments (a–c–e) was with 162.4°±6.5° again larger than for heathland sheep.

### Endoscopy

**Figure 2:**
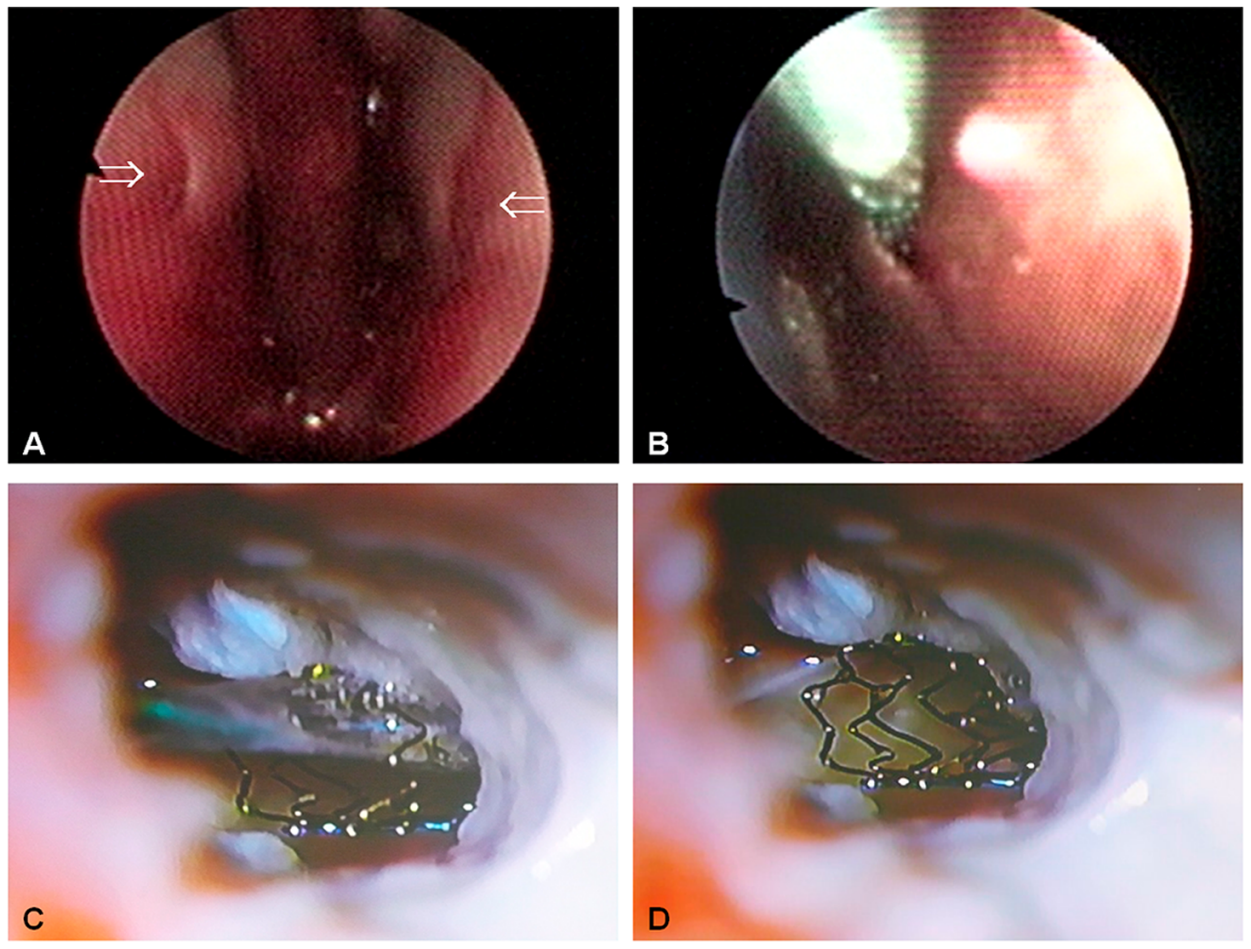
Insertion of a coronary stent into the Eustachian tube of heathland sheep. **A**: View through the bronchofiberscope at both ostia pharyngea (⇒). **B**: Insertion of a stent through the working channel of the fiberscope. **C**: Microscopic view of the middle ear. The stent can be moved forward through the isthmus into the middle ear. **D**: After dilatation of the stent and retraction of the catheter the stent remains in position (view from the middle ear).

Heathland sheep have a smaller head and shorter nose compared to blackface sheep. The best approach to the ostium pharyngeum was through the nose for both strains. When inserted through the nostril, it was possible to visualize both pharyngeal ostia with the bronchofiberscope (Fig. 2A). The flexible miniature optic could be moved forward through the working channel into the contralateral ET. Nevertheless with the current equipment it was only possible to investigate the first few millimeter of the ET as touching the mucosa inside the slightly curved tube always obstructed the view through the miniature optic.

### Stent insertion

**Figure 3:**
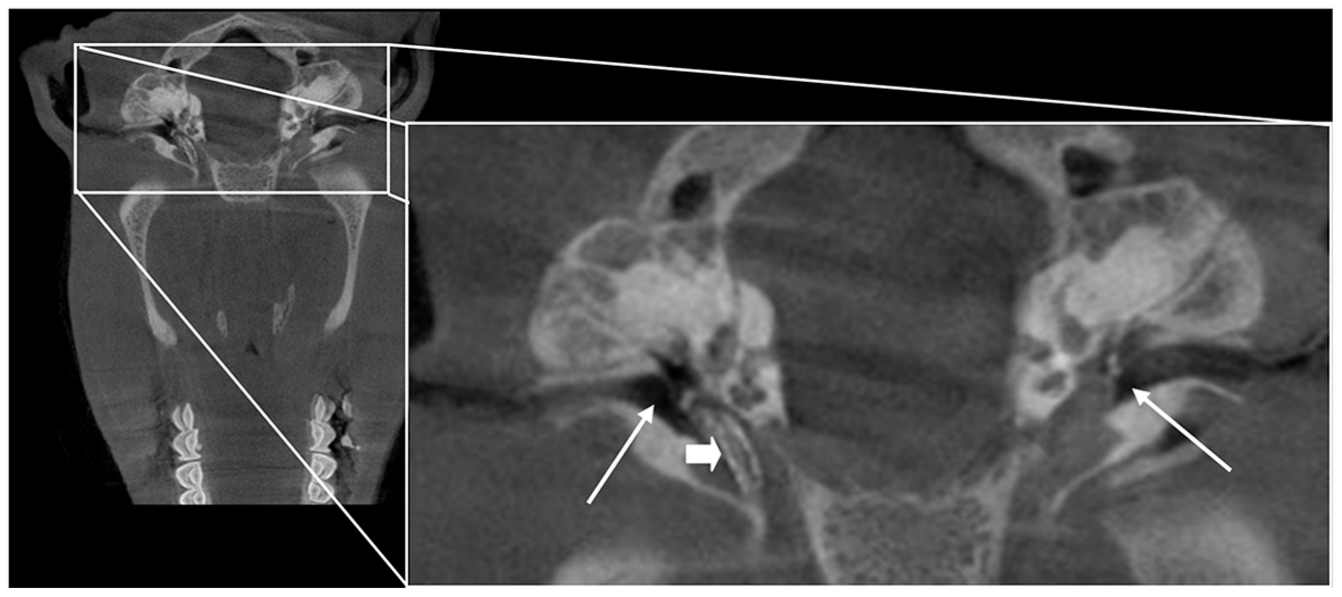
DVT image of a blackface sheep after insertion of a 9 mm stent into the tube. Position of the middle ears are marked by thin arrows (↑) and the stent is indicated by a block arrow (⇒).

After placing a guide wire through the working channel of the bronchofiberscope inside the ET a stent could be inserted (Fig. 2B). It was possible to move it forward through the isthmus into the middle ear (Fig. 2C) even though this deep insertion is not intended for *in vivo* experiments. In this cadaver study, the position of the stent was verified with a microscope through the opened middle ear. The stent was inflated successfully and stayed inside the tube after removing guide wire and balloon catheter (Fig. 2D). Examination of the stented cartilaginous part of the ET revealed that the miniature optic gets stuck at the struts of the stent. Proper positioning of the metal stent could be verified by DVT (Fig. 3).

## Discussion

The aim of the current study was to investigate whether the sheep could be an appropriate animal model to test surgical treatments for Eustachian tube disorders in humans. As the middle ears of pig and sheep are considered to be comparable to the human middle ear [19] and the pig was reported to have a pure cartilaginous tube [21], two different breeds of sheep (heathland sheep and blackface sheep) were investigated in terms of dimensions of the ET and possible approaches for a stent insertion.

To evaluate the dimensions of the ET, the tubes were filled with silicone enriched with BaSO_4_. Using this approach the tubes could be distinguished and segmented in DVT data sets. Sometimes air bubbles were trapped inside the silicone. Many small air bubbles became obvious after maceration especially in blackface sheep whereas the silicone surface of samples from heathland sheep appeared smoother. Due to the use of an accelerator for curing, degassing of the silicone before use was omitted because of the short curing times. This might have contributed to differences between different experimental days. On the other hand the use of the accelerator was mandatory to assure complete curing inside the tube at room temperature at a reasonable time. When the air bubbles were at the surface of the silicone these might have lead to an underestimation of tube diameters at that point and therefore can influence also the position of the isthmus. This can contribute to the large variation in the distances from the middle ear to the isthmus in blackface sheep. Here, this distance varies between 0.8 and 10.7 mm.

When investigating the dimensions of the tubes from DVT data, the general settings were kept constant but for segmentation the thresholds had to be reduced from 75% (heathland sheep) to 50% (blackface sheep) in order to clearly distinguish between the silicone and surrounding tissue. The reason for this change was that the bone appeared more intense (background) in DVT data from blackface sheep. One explanation for this can simply be the larger head (more bone) of the blackface sheep. If these different thresholds have an influence on the data, we expect a slight underestimation in tube diameters of blackface sheep compared to heathland sheep. This seems to be supported by the smaller differences in diameter between DVT data and data from the macerated silicone model and the larger diameter at the ostium pharyngeum in the silicone model of blackface sheep.

To determine the dimensions of the tube in the segmented data the ideal solution would be a trajectory that follows the curvature of the tube always in its centre from the middle ear to the ostium pharyngeum. To approximate this ideal situation, each tube was divided in 4 sections with an equal number of images. The start and end points of the trajectories in each section were positioned in the center of the tube. Such it could be assured that all trajectories were always inside the tube and by measuring the diameter perpendicular to it, the influence of the curvature on the values was kept to a minimum.

By maceration of the surrounding bone and tissue, a silicone model of each filled tube was received. Comparison of the measures of the silicone models with the DVT data resulted with the exception of the diameter of the ostium pharyngeum in blackface sheep in longer distances and smaller diameters in the silicone models. For the latter there are two possible explanations: i) due to the intense signal of the BaSO_4_ filled silicone in the data sets, outshine effects might have led to the measurement of larger diameters and additionally ii) the maceration process might also affect the silicone especially with the BaSO_4_ filler. Furthermore, the silicone appeared to be very soft and sticky after maceration. Therefore, the material could also unintendedly be slightly compressed during the measurements with the caliper. Additionally, if there is a long thin section of the model in the isthmus region determination of the position of the smallest diameter of the model bears some uncertainties as the possible resolution for these measurements is much higher in DVT data sets. In the case of air bubbles, these might have affected position and diameter of the isthmus in both the DVT data and the silicone model. Both effects contributed to the large variations in the position of the isthmus. Measuring the diameters from the scanned data seems therefore to be more reliable also in regard to the position at which the diameter is determined. The diameters in the cartilaginous part close to the ostium pharyngeum do not resemble the maximum diameters of the ET as during filling it was not attempted to maximally expand the tube but just to fill the existing open structure. The lengths of the different parts of the ET were larger in the silicone models. In both, the data sets and the silicone models, it was challenging to determine the start and end points of the tube even though the silicone that was leaking out of the ostium was measured and subtracted from the segmented data or the total length of the silicone models. Furthermore, due to the curvature of the ET, the lengths in the DVT data were considered to be more accurate. To measure the lengths in the silicone models, these had to be straightened or the influence of the curvature would not be considered. Both would introduce more uncertainties to the values for the silicone model than the segmented tube.

**Figure 4:**
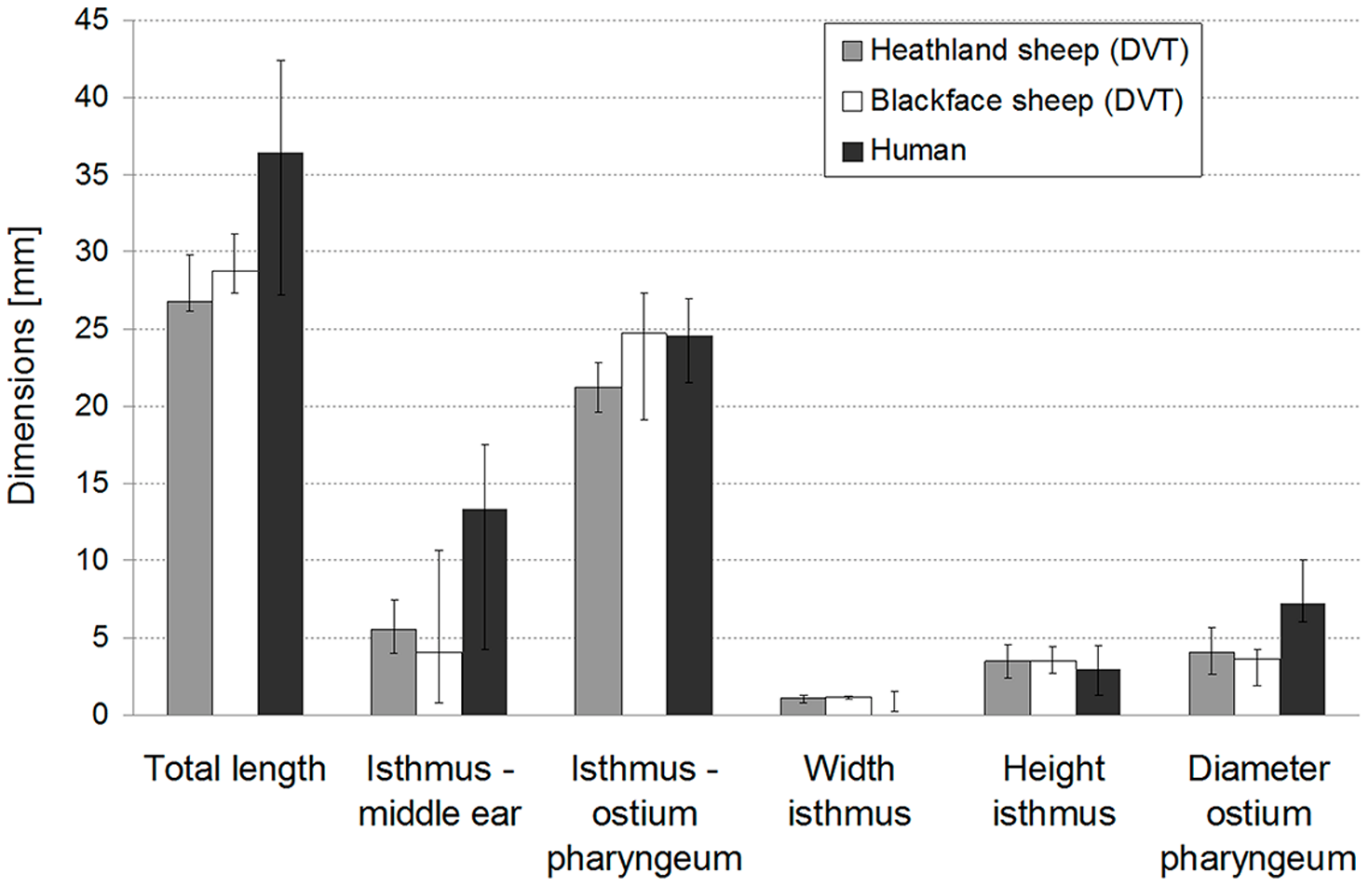
Comparison of the tube dimensions of heathland and blackface sheep with human data from other publications (see also **Table 3**). Mean values as well as maximum and minimum are presented. For the width of the human isthmus, no mean data were available; such only maximum and minimum values are presented.

When comparing both breeds, the main difference with more than 3 mm (DVT data) was in the distance from the isthmus to the ostium pharyngeum (Table 1). As this resembles the cartilaginous part of the tube, this length together with the diameter of the isthmus is the most important measure for a comparison with the human ET because in the majority of cases pathologic changes can be found in the cartilaginous portion of the tube [26]. As summarized in Fig. 4, both match the reported dimensions of the human ET very well. The presented human values are based on the literature data summarized in Table 3. Therefore, according to our investigation, the dimensions of the ET especially of the blackface sheep are very well comparable to human dimensions. The curvature of the tube in both strains of sheep decreases from the ostium pharyngeum to the middle ear. This might be of interest when developing stents for the ET to treat middle ear ventilation disorders. Data for humans on this curvature are currently not available. It is only reported that the angle at the isthmus varies between 160° and 180° depending on the tension of the M. tensor veli palatini [27]. Therefore any comparison on this can currently not be done between sheep and human.

The tube of both strains of sheep was accessible via a bronchofiberscope through the nose. A coronary stent (O.D. 0.9 mm) could be advanced through the ET into the middle ear and fixed at the isthmus through the working channel of the endoscope. Even though these were cadaver implantations, no visible damage occurred. This implies that this approach can also be used for *in vivo* investigations. As there is no online control about the insertion depth of the stent in the ET, the most promising orientation might be special markers on the balloon and its lead. Due to the dimension of stent and tube, it should then be possible to ensure proper positioning of the stent inside the tube.

Endoscopic implantation of the stent from the nasopharynx provides an approach to treat middle ear ventilation disorders without opening the tympanic membrane. This approach is currently only taken for balloon dilatation of the ET in patients [12] whereas all earlier implants like gold wires [8] or early stents [6] were inserted from the ostium tympanicum.

In summary, the dimensions especially of the tube of blackface sheep match very well the dimensions of the human ET. Additionally, the tube can be endoscopically investigated through the nose of the sheep and it is possible to implant a stent through the ostium pharyngeum and fix it at or behind the isthmus. Therefore we conclude that the sheep is a suitable large animal model for treatments of ventilation disorders of the middle ear.
